# Epidemiology and acquisition of extended-spectrum beta-lactamase-producing *Enterobacteriaceae* in a septic orthopedic ward

**DOI:** 10.1186/2193-1801-2-91

**Published:** 2013-03-08

**Authors:** Americo Agostinho, Gesuele Renzi, Thomas Haustein, Ghislaine Jourdan, Chantal Bonfillon, Mathieu Rougemont, Pierre Hoffmeyer, Stephan Harbarth, Ilker Uçkay

**Affiliations:** 1 Infection Control Program, University of Geneva Hospitals and Faculty of Medicine, Geneva, Switzerland; 2 Laboratory of Bacteriology, University of Geneva Hospitals and Faculty of Medicine, Geneva, Switzerland; 3 Orthopaedic Surgery Service, University of Geneva Hospitals and Faculty of Medicine, Geneva, Switzerland; 4 Service for Employees, University of Geneva Hospitals and Faculty of Medicine, Geneva, Switzerland

**Keywords:** ESBL, Septic orthopedics, Patients, Healthcare workers, Antibiotics

## Abstract

Wards cohorting infected orthopaedic patients may be particularly prone to transmitting extended-spectrum beta-lactamase-producing Enterobacteriaceae (ESBL-E). We analyze their epidemic pattern by performing molecular typing of ESBL-E isolated from patients and healthcare workers (HCW) from our septic ward. Between March 2010 and November 2011, 186 patients were admitted. Among 565 anal swabs, ESBL-E were detected in 204 samples from 45 patients, suggesting prolonged carriage in affected patients. Among 25 cases with identical ESBL-E species and positive epidemiological links, only 9 were really attributable to our service. We also screened 41 healthcare workers (HCW) on 49 occasions during the study period. Six samples (13%) were positive. None of the ESBL-E detected in HCW were related to any of the patient isolates. Among 60 environmental samples taken at the peak of the epidemic none revealed ESBL-E. We conclude that HCW also were anal carriers of ESBL-E, however the ESBL- strains from the HCW were not the same strains isolated from patients in the septic ward. Moreover, the epidemiological attribution of ESBL by simple vicinity, timing, and species identification might grossly overestimate transmission within a given unit.

## Introduction

Orthopaedic surgical site infections are often associated with substantial morbidity and exorbitant costs, and are challenging to treat, especially in case of multi-resistant pathogens or presence of implants (U&çkay et al. 2010; Martinez-Pastor et al. 2010). Thus, to protect uninfected implant patients, many centres cohort infected patients in specialized septic wards, although the scientific benefit for this practice is lacking.

Extended-spectrum beta-lactamase producing Enterobacteriaceae (ESBL-E) are emerging worldwide in hospitals (Fankhauser et al. 2009) and in the community (Kader && Kamath 2009). Septic wards are an ideal place for their proliferation (Martinez-Pastor et al. 2010; Uçkay et al. 2009a) due to the selective pressure exerted by intensive use of penicillins and cephalosporins, long hospital stays with intensive close nursing and physiotherapy for multi-morbid and immobile patients, high prevalence of open wounds, decubitus ulcers or external fixation devices, and lack of established decolonization protocols for ESBL-E.

In this study, we assessed the epidemiology of ESBL-E in our septic orthopedic ward by typing and individual epidemiological attribution of the source of acquisition. We also examined the possible role of healthcare workers (HCW) with anal ESBL-E colonization on patients in this ward.

### Setting

The University of Geneva Hospitals is a 2200-bed tertiary healthcare center. The Orthopedic and Traumatology Service has 132 acute care beds with a septic orthopaedic ward of 24 beds, 4 surgeons, 19 nurses and 7 auxiliary nurses, 2 physiotherapists, 1 cleaning specialist, 1 infectious diseases physician with specialisation in infection control and antibiotic policy (Uçkay et al. 2009b), and a wound care service (5 nurses) for 5,000 annual ambulatory consultations.

### ESBL-E policy on the ward

Since 2009, admissions on the septic ward with a probable hospital stay beyond 3 days have been screened for ESBL-E (1 anal swab); unless the patient is known to be positive or is transferred from another ward where screening has been performed in the last few days. Patients with a history of ESBL-E carriage are flagged in a computerized alert system and put in pre-emptive contact isolation. This attitude also applies to patients transferred from abroad (Fankhauser et al. 2009), roommates of index patients (Fankhauser et al. 2009) or during outbreaks, when weekly patient screenings are performed. Routine antibiotic prophylaxis for orthopedic surgery does not cover ESBL-E.

ESBL-E carriers remain contact-isolated throughout their hospital stay with no decolonization performed. Their rooms, toilets and floors are cleaned with 80% magnesium monoperoxyphthalate hexahydrate (Dismozon®, Bode Chemie, Hamburg, Germany) or troclosene 2.5% during outbreaks. Hand hygiene is continuously promoted according to hospital policy. Observed adherence to hand hygiene recommendations (Sax et al. 2007) ranges between 56% and 80% of indicated opportunities. Voluntary and anonymzed ESBL-E screenings are offered to HCW.

### Microbiological procedures

On admission, anal ESBL-E carriage is detected by a commercial chromogenic agar (chromID ESBL medium, bioMérieux, Marcy l’Etoile, France). For this study we typed ESBL-E isolates using repetitive sequence-based PCR (DiversiLab®, bioMérieux, Marcy l’Etoile, France) (Brolund et al. 2010). ESBL isolates with a similarity of ≥ 97.5% were considered indistinguishable.

## Results

Between March 2010 and November 2011, 186 patients were admitted from the community and 1335 transferred from other institutions, totaling 12,401 patient-days with an average length of hospital stay of 27 days. Bed occupancy averaged 83%.

Among 565 anal swabs, ESBL-E was detected in 204 samples from 45 patients, suggesting prolonged carriage in affected patients. In six patients, two different ESBL-E strains were detected, and 3 patients carried three distinct isolates. Among the 45 positive patients, 29 (64%) were detected during the first three days of admission, the remainder after a median of 13 days of hospitalization, range 7–52 d). At the time of sampling, 26 patients received antibiotic therapy without clinical activity against their respective ESBL-E; a further seven patients were treated with antibiotics which their ESBL-E strains were susceptible to *in vitro* (carbapenems or quinolones). Most positive patients were asymptomatically colonized with ESBL-E. Two patients had arthroplasty infections due to ESBL-E, of which one was acquired on our ward. No urinary tract, other implant infections or soft tissue infections due to ESBL were witnessed. The isolated ESBL-E were *E. coli* (n = 39), *Enterobacter* spp (8), *Citrobacter* spp (3), *Klebsiella pneumoniae* (1), *Morganella morganii* (1), and *Proteus vulgaris* (1).

We also screened 41 HCW on 49 occasions during the study period. Six samples (13%) were positive. Among 60 environmental samples taken at the peak of the epidemic (room floors, beds, curtains, tables, doors, offices, computers, telephones, kitchen, physiotherapy material, and toilets), none revealed ESBL-E.

### Genotyping

Epidemiological attribution alone was considered as insufficient to prove nosocomial transmission; e.g., two epidemiologically related patients with an *E. coli* carriage may reveal in fact different isolates when typing is performed. On the other hand, two identical ESBL-E strains found on admission may occur in patients that were never hospitalized in our service. These latter cases were regarded as community-acquired, although there might have been a cross-transmission in another service or another hospital before.

**Figure 1:**
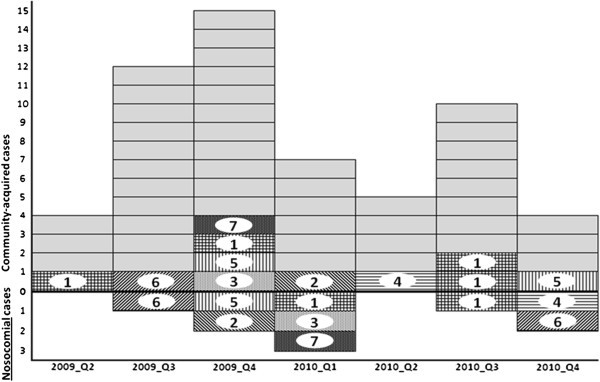
**Epidemic curve of ESBL anal colonization in our septic orthopaedic ward.** Horizontal axis: Study years stratified in quarters. Vertical axis: Number of positive ESBL isolates on the septic ward. The zero lines divide community-acquired cases (above) from nosocomially acquired cases (below). Grey cases are isolates without genotypic relation to other isolates. Genotypically identical strains are marked with numbers (for example isolate 5 occurs three times).

Among the 45 positive patients, we identified few clusters of likely nosocomial transmissions in our ward (9 patients; Figure 1). The rest were either unrelated or lacked an epidemiological relationship within the orthopedic service, since these ESBL-E were identified on admission in patients that had never been hospitalized before in our unit. Without molecular typing, we would have recorded 25 presumed “nosocomial” cases attributed to our ward. None of the ESBL-E detected in HCW were related to any of the patient isolates.

## Discussion

We report three interesting findings.

First, HCW may also be anal carriers, but their strains might be different from the patients’; even if these reveal long hospital stays. While no study so far has investigated prevalence or incidence of anal ESBL-E carriage among HCW, it is not *per se* surprising that HCW become anal carriers, as also a substantial part of general population may be carriers nowadays (Kader && Kamath 2009). In an Egyptian study, ESBL-E hand colonization among HCW was 2% (Rahman et al. 2010). Unfortunately, despite 13% prevalence among voluntarily screened HCW, we could not explain why there was no matching at all with the patients’ ESBL-E epidemic.

Second, among 25 cases with identical ESBL-E species and positive epidemiological links, only nine were attributable to our service. This underlines that epidemiological attribution of ESBL-E by simple vicinity, timing, and species identification might grossly overestimate transmission within a given unit.

Finally, the rationale for systematic ESBL-E screening (either on admission or periodically) in an orthopaedic ward can be questioned. Although 565 samples have been performed on our septic ward alone, only one nosocomial ESBL-E infection was attributed to our unit. While this relation might appear to lack cost-effectiveness, a regular analysis regarding the accuracy of ESBL-E detection in orthopaedic surgery is lacking in the literature. Epidemiologically speaking, ESBL-E are a rare cause of orthopaedic site infections in resource-rich settings (Martinez-Pastor et al. 2010; Uçkay et al. 2009a).

Our study has limitations. It is a single-centre study with small sample size and no systematized screening of patients or HCW. In Switzerland, a non-voluntary sampling of HCW is impossible. The DiversiLab system may be less accurate than other techniques such as multilocus sequence typing (Brolund et al. 2010). Likewise, the point at which genetic relatedness is determined remains arbitrary. We have chosen a very conservative threshold of 97.5%.
